# Prevalence and characterization of West Nile virus infections among people living with HIV in Northern Italy: a sero-repository analysis

**DOI:** 10.1007/s15010-026-02919-3

**Published:** 2026-08-11

**Authors:** Giorgio Tiecco, Concetta Castilletti, Alessandro Pavesi, Silvia Accordini, Natasha Gianesini, Jacopo Logiudice, Rebeca Passarelli Mantovani, Benedetta Rossi, Lorena Maria Chesini, Stefano Calza, Elena Raffetti, Federico Giovanni Gobbi, Lina Rachele Tomasoni, Francesco Castelli, Eugenia Quiros-Roldan

**Affiliations:** 1 Department of Clinical and Experimental Sciences, Unit of Infectious and Tropical Diseases, University of Brescia and ASST Spedali Civili di Brescia, Brescia, 25123 Italy; 2 Department of Infectious-Tropical Diseases and Microbiology, IRCCS Ospedale Sacro Cuore Don Calabria, Negrar di Valpolicella, Verona, Italy; 3 Unit of Infectious and Tropical Diseases, ASST Spedali Civili di Brescia, Brescia, 25123 Italy; 4 Unit of Biostatistics and Bioinformatics, Department of Molecular and Translational Medicine, University of Brescia, Brescia, Italy; 5 Department of Global Public Health, Karolinska Institutet, Stockholm, Sweden

**Keywords:** West Nile virus, WNV, Italy, Serology, Prevalence

## Abstract

**Background:**

West Nile virus (WNV) is increasingly reported in Italy, yet its population prevalence remains uncertain. People living with HIV (PLWH) may be at increased risk of WNV severe manifestations, including neuroinvasive disease. This study assessed the seroprevalence and factors associated with WNV seropositivity among PLWH in a large Northern Italian cohort.

**Methods:**

This retrospective single-center study included all PLWH followed at the Brescia HIV Outpatient Clinic with at least one serum sample collected in 2022 and stored in the institutional sero-repository. Samples were screened for WNV antibodies using ELISA; reactive or borderline samples were confirmed by immunofluorescence assay for IgG and IgM and further characterized by microneutralization assays to differentiate WNV from USUV infection. Associations with seropositivity were evaluated using univariate logistic regression.

**Results:**

Among 2,843 PLWH, 76.2% were male, the mean age was 54.1 (± 10.5), and 87.2% were of Italian origin. Most participants had virologically suppressed HIV infection and were receiving stable antiretroviral therapy (94.2%). WNV serological screening by ELISA identified IgG positivity in 176 participants (6.2%). Of these, 166 tested positive by immunofluorescence assay. Neutralization assays subsequently confirmed WNV seropositivity in 86 participants (3.0%), USUV seropositivity in 12 (0.6%), and dual WNV/USUV neutralizing antibody positivity in 6 (0.2%). No neuroinvasive diseases were reported. Compared with seronegative individuals, seropositive participants were less frequently male (*p* = 0.001), younger (*p* < 0.001) and differed significantly by geographic origin (*p* < 0.001), with a higher prevalence among PLWH born outside Europe. In logistic regression analyses, geographic origin was strongly associated with seropositivity: participants from Africa (OR 21.22, 95% CI 12.49–36.03), Asia (OR = 23.31, 95% CI: 10.19–53.31), and the Americas (OR 19.16, 95% CI 9.45–38.83) had markedly higher odds compared with participants of Italian origin (all *p* < 0.001).

**Discussion:**

This is, to our knowledge, the first study evaluating WNV seroprevalence among PLWH. Seroprevalence was higher than that reported in comparable European blood donor populations. In this largely virologically suppressed, stably treated outpatient cohort, no severe manifestations were observed and symptoms were infrequently recalled.

**Supplementary Information:**

The online version contains supplementary material available at https://doi.org/10.1007/s15010-026-02919-3.

## Background

West Nile Virus (WNV) is an *orthoflavivirus* with a single-stranded RNA genome, transmitted primarily by mosquitoes of the *Culex* genus, with birds serving as the main reservoir [1]. Human infection is often asymptomatic or manifests as West Nile Fever (WNF), while a minority of cases develop severe neuroinvasive disease (WNND), particularly in immunocompromised or elderly individuals [2]. Human WNV seroprevalence varies widely, ranging from 0.7% to 52%, depending on geographic location and methodological differences, particularly owing regarding cross-reactivity among *orthoflaviviruses* [3]. A recent meta-analysis that restricted pooled estimates to confirmed WNV infections verified by neutralisation assays reported a global seroprevalence of 3.6% (95% CI: 2.0–6.5), with substantial regional heterogeneity, though this likely underestimates the true burden due to underreporting and limited diagnostic capacity, particularly in low-resource settings [3, 4].

In recent decades, climate change, altered bird migration, and high vector density have contributed to the increasing incidence of WNV in Southern Europe, including Italy [5]. In Italy, WNV was first detected in horses in 1998 [6], with the first human outbreak reported in 2008 in the Po valley of Emilia-Romagna and Veneto regions [7, 8], followed by sustained annual circulation with an average of approximately 60 laboratory-confirmed cases annually, predominantly in northern regions, and major epidemic peaks in 2018 and 2022 [9]. In 2022, more than 300 cases of WNND were reported, with a case fatality rate exceeding 10%, highlighting the growing public health impact [10]. Usutu virus (USUV), which co-circulates with WNV, has been detected in Italy since 1996 and, under surveillance since 2017, has been associated with sporadic human infections, including neuroinvasive disease [11]. USUV is an emerging arbovirus in Europe, where repeated introductions from Africa, followed by local evolution and dispersal through migratory birds, have contributed to its establishment and geographic expansion [12, 13]. Epizootics among wild and captive birds and the detection of asymptomatic and acute infections in blood donors highlight the increasing potential for human exposure and the importance of blood safety surveillance [14–16]. Current management relies solely on supportive care, as no vaccines or antiviral therapies are available [17], and the true population-level prevalence remains poorly defined due to the high proportion of asymptomatic infections, despite the implementation since 2020 of a national One Health surveillance plan integrating WNV and USUV monitoring, risk-based regional stratification, and early intervention strategies [9].

Immunocompromised individuals are considered at increased risk of developing severe manifestations of WNV infection and people living with HIV (PLWH) may therefore represent a potentially vulnerable population [18, 19]. However, clinical evidence of WNV infection in PLWH remains limited and is largely confined to case reports. WNV infection in PLWH is nonetheless of interest, as several antiretroviral agents seem to have in silico and in vitro activity against arboviruses, including WNV, such as the non-nucleoside reverse transcriptase inhibitor (NNRTI) etravirine or rilpivirine, which inhibits WNV RNA-dependent RNA polymerase [20, 21], and doravirine, a next-generation NNRTI with improved tolerability and enhanced blood–brain barrier penetration that has been proposed as a potential candidate based on structural similarity to rilpivirine [22, 23].

This study aims to assess the seroprevalence and factors associated with WNV seropositivity among PLWH in our cohort using serum samples collected in 2022 and stored in a large sero-repository.

## Methods

### Study design and participants

This observational, retrospective, single-center study included all PLWH enrolled in the MASTER cohort with at least one serum sample collected in 2022 and stored in the sero-repository of the Unit of Infectious and Tropical Diseases, ASST Spedali Civili of Brescia. Human serum samples were collected between January 1 and December 31, 2022, from outpatients attending the HIV clinic at the same institution. Eligibility criteria were age ≥ 18 years, enrolment in the MASTER cohort, and availability of at least one archived serum sample from 2022. No exclusion criteria were applied.

### Laboratory testing

All serum samples were centralized and analysed at the Reference Laboratory of IRCCS Ospedale Sacro Cuore Don Calabria, Negrar, Italy. WNV-specific IgG antibodies were initially screened using a commercial enzyme-linked immunosorbent assay (ELISA) method (Anti–West Nile Virus ELISA IgG, Euroimmun AG, Lübeck, Germany), performed according to the manufacturer’s instructions. Results were expressed as a ratio between the sample optical density and calibrator values. According to the manufacturer’s instructions samples with a ratio of less than 0.8 (< 0.8) were considered negative, samples with a ratio between 0.8 and 1.1 as borderline, and samples with a ratio of 1.1 or greater (≥ 1.1) were considered positive. To rule out nonspecific results, all ELISA-positive and borderline samples underwent a first confirmatory test by indirect immunofluorescence assay (IFA) (Flavivirus Mosaic 1 IIFT, Euroimmun AG) for WNV IgG antibodies. In addition, all samples that tested positive for WNV IgG by IFA were further analysed for the presence of specific IgM to identify recent infections. Screening dilution was 1:20; titers < 1:20 were considered negative and ≥ 1:20 positive. IgG- and IgM-positive samples positive by IFA were further evaluated by differential microneutralization assays (MNTA) to differentiate WNV (WNV-2 clinical isolate kindly provided by Tiziana Lazzarotto, Bologna, Italy) from USUV (Usutu strain ENT MP 1626 (VIR-1892), American Type Culture, Manassas, Virginia, USA) infection, given the known serological cross-reactivity among flaviviruses [24] (Figure S1). Briefly, serum samples were heat-inactivated at 56 °C for 30 min and titrated in duplicate in 7 twofold serial dilutions (starting dilution 1:10) in serum-free maintenance medium. Equal volumes (50 µL) of virus preparations containing 100 TCID_50_ of WNV or USUV and serum dilutions were mixed and incubated at 37 °C for 30 min. Subsequently, 96-well tissue culture plates with sub-confluent Vero E6 cell (ATCC, Manassas, Virginia, United States) monolayers were incubated at 37 °C and 5% CO_2_ with 100 µL/well of each virus/serum mixture in duplicate. Each serum sample was checked for intrinsic cytotoxicity in the same plate. In each MNTA session, the virus control was back-titrated to verify the correct TCID^50^ inoculum dose. To standardize inter-assay procedures, negative and positive control samples with high (1:160) and low (1:40) neutralizing activity were included in each assay session. After 48–72 h, microplates were examined under light microscope for the presence of cytopathic effect (CPE). The highest serum dilution that inhibited at least 90% of the CPE was defined as positive for the presence of neutralizing antibodies (nAbs). Neutralizing antibodies (NAb) against WNV and USUV were assessed using a screening dilution of 1:10; titers < 1:10 were considered negative and ≥ 1:10 positive.

### Data collection

Patient medical records were reviewed to obtain demographic information, including age, gender, ethnicity, nationality, and domicile. HIV-related data included year of diagnosis, antiretroviral therapy (ART) status, treatment interruptions exceeding one year, discontinuities in the last five years, and viro-immunological profile (HIV-RNA, CD4 and CD8 cell counts and percentages, and CD4/CD8 ratio) at the time of serum sampling. Participants were classified as residing in a WNV/USUV-circulating area if their domicile fell within territories designated as at risk for WNV/USUV infection in the 2022 Regional (Lombardy, Italy) surveillance report [25], based on documented WNV circulation in birds, horses, mosquito vectors, and/or humans. For PLWH with confirmed WNV or USUV infection by neutralization testing, additional clinical and epidemiological information was collected by the treating physician during routine six-month follow-up visits. Travel-related variables included travel within the previous five years to areas classified as at risk for WNV transmission according to the Centers for Disease Control and Prevention [26]. In addition, the occurrence during travels or summer 2022 of fever, headache, rash, or arthralgia was specifically recorded. All anonymized data were systematically recorded in a dedicated electronic database (REDCap) [27].

### Ethics

The study protocol was approved by the Lombardy Region Ethics Committee (protocol number 3674/24) and conducted in accordance with the ethical standards of the Helsinki Declaration (1975, revised in 2013). Written informed consent has been obtained from each subject.

### Statistical analysis

Categorical variables were summarized as counts and percentages. Continuous variables (CD4 cell count nadir, current CD4 and CD8 cell count, and CD4/CD8 ratio) were summarized using means with standard deviations (SD) and medians with interquartile ranges (IQR), as appropriate. Missing data were reported for each variable and were not imputed. Comparisons between participants with and without positive neutralization assays (WNV and/or USUV) were performed using Pearson’s χ² test for categorical variables, with Monte Carlo simulated p-values (2,000 replicates). Continuous variables were compared using the Wilcoxon-Mann-Whitney rank-sum test. Associations between demographic, clinical, and immunological variables and seropositivity (coded as binary variable) were evaluated using univariate logistic regression models. Separate models were fitted for (i) WNV neutralization positivity, (ii) USUV neutralization positivity, and (iii) combined WNV and/or USUV neutralization positivity. Results were reported as Odds ratios (ORs) with 95% confidence intervals (95% CIs). Due to quasi-complete or complete separation in some categorical predictors (e.g., geographic origin categories with zero events), we adopted Firth’s bias-reduced penalized-likelihood logistic regression [28]. Predicted probabilities with 95% CIs were derived from fitted models when relevant. Variables associated with seropositivity at the univariate analysis and clinically relevant covariates were included in multivariable logistic regression models to identify factors independently associated with WNV and/or USUV seropositivity. As a sensitivity analysis, the multivariable model was repeated after restricting the analysis to participants of Italian origin. All statistical analyses were conducted using R statistical software (R Foundation for Statistical Computing, Vienna, Austria, version 4.5.2) [29]. All tests were two-sided, assuming a 5% significance level.

## Results

A total of 2,843 PLWH were included in the analysis. Most were male (76.2%), with a mean age of 54.1 (± 10.5) years, and of Italian origin (87.2%). Overall, 37.3% of participants resided in municipalities classified as WNV-circulating areas in 2022. Foreign nationality (239, 23%) was more frequent (*p* < 0.001) among PLWH living in WNV-circulating municipalities compared with Italian nationals (820, 77%). The mean CD4 cell count at sampling was 801 (± 369.5) cells/mm³, and the mean CD4/CD8 ratio was 1.01 (± 0.59). Most participants had undetectable (viral load < 20 copies/mL) plasma HIV-RNA (94.2%) and were on stable ART (94.2%), with no evidence of treatment discontinuation within the preceding 12 months (Table 1).

**Table 1:** Demographic, clinical, and virological characteristics of the study population

*N*, (%)	2,843 (100)
Demographics	
Male, *n* (%)	2,166 (76.2%)
Age, mean (SD)	54.1 (± 10.5)
Geographic origin	
Italy, *n* (%)	2,478 (87.2%)
Africa, *n* (%)	253 (8.9)
Other, *n* (%)	112 (3.9)
Residence data	
Living in a 2022 WNV-circulating area, *n* (%)	1061 (37.3)
Antiretroviral therapy (ART)	
On stable ART*, *n* (%)	2,678 (94.2%)
Immunological profile	
CD4 cell count nadir (cells/mcL), mean (SD)	238.9 (191.6)
Current CD4 cell count (cells/mcL), mean (SD)	801.1 (369.5)
Current CD8 cell count (cells/mcL), mean (SD)	946.0 (484.0)
Current CD4/CD8 ratio, mean (SD)	1.01 (0.59)
WNV ELISA assays results	
IgG positive, *n* (%)	143 (5.0)
IgG borderline, *n* (%)	33 (1.2)
WNV Immunofluorescence assays results	
IgG + IgM-, *n* (%)	160 (5.6)
IgG + IgM+, *n* (%)	6 (0.2)
Neutralization assays results	
Confirmed WNV or USUV cases (*), *n* (%)	98 (3.4)
WNV positive, *n* (%)	86 (3.0)
USUV positive, *n* (%)	18 (0.6)
WNV and USUV positive, *n* (%)	6 (0.2)
Anamnestic data and symptoms among confirmed WNV or USUV cases (*)	
History of travel (self-reported), *n* (%)	44 (44.9)
Symptoms reported during travel, *n* (%)	3 (3.1)
Symptoms reported during summer, *n* (%)	2 (2.0)

*WNV* West Nile Virus, *USUV* Usutu virus, *ELISA* enzyme linked immunoadsorbed assay, *SD* standard deviation. *On stable ART, defined as no treatment discontinuation exceeding one year since ART initiation; (*) WNV or USUV case defined as neutralization assays positivity

WNV serological screening by ELISA identified IgG positivity in 143 participants (5.0%) and borderline reactivity in 33 (1.2%). Among ELISA-reactive samples subsequently tested by immunofluorescence, 160 (90.9%) were IgG⁺/IgM⁻ and 6 (3.4%) were IgG⁺/IgM⁺ (Table 1). Neutralization assays confirmed WNV infection in 86 individuals (3.0%) and USUV infection in 12 (0.6%), 6 (0.2%) showed neutralizing antibodies against both WNV and USUV and were classified as dual-seropositive (Fig. S1). Of the participants with confirmed WNV and/or USUV infection, 44 (44.9%) reported a history of travel to areas classified as at risk for WNV/USUV transmission within the previous 5 years. Non-specific symptoms during travel were reported by 3 individuals (3.1%), primarily fever and headache, and 2 (2.0%) reported symptoms during the summer season. No definite association with WNV infection could be established. No cases of neuroinvasive disease were reported (Table 1). Similarly, no symptoms were reported among the six IgG⁺/IgM⁺ individuals, who were predominantly male (67%), with a mean age of 55.7 (± 7.66) years, and on stable ART (83%), with preserved current CD4 cell counts (mean 937.7 cells/µL). Of these, four (67%) had neutralization-confirmed WNV infection and two (33%) had neutralization-confirmed USUV infection. Moreover, 3 (50%) of those samples were collected during the national WNV transmission season (May–October). Detailed demographic, clinical, and virological characteristics are extensively provided in Table S1.

When comparing individuals with positive neutralization assays for WNV or USUV to seronegative PLWH (Table 2), positive individuals were less frequently male (*p* = 0.001), younger (*p* < 0.001), and differed significantly by geographic origin (*p* < 0.001). Seropositive participants were more frequently from Africa, the Americas, and Asia, whereas Italian origin was more common among seronegative individuals (84% vs. 28%). A lower proportion of seropositive individuals were on stable ART compared with seronegative participants (*p* = 0.043). No significant differences were observed in CD4 cell count nadir, current CD4 and CD8 cell count, or CD4/CD8 ratio between groups.

**Table 2:** Comparison of neutralization assay results between WNV or USUV cases and seronegative PLWH

Neutralization assays	Positive	Negative	*P* value
*N* (%)	98 (100)	2,745 (100)	
Demographics			
Male, *n* (%)	56 (57%)	2,110 (76.5%)	**0.001**
Age, mean (SD)	50.3 (± 10.4)	54.2 (± 10.4)	**< 0.001**
Geographic origin			**< 0.001**
Italy, *n* (%)	27 (28%)	2,451 (89.3)	
Africa, *n* (%)	44 (45%)	209 (7.6%)	
Other, *n* (%)	27 (28%)	85 (3.1%)	
Antiretroviral therapy (ART)			**0.043**
On stable ART*, *n* (%)	81 (85%)	2,597 (94.6%)	
Immunological profile			
CD4 cell count nadir (cells/mcL), mean (SD)	248.52 (189.32)	238.63 (191.66)	0.5
Current CD4 cell count (cells/mcL), mean (SD)	792.59 (422.81)	801.34 (367.63)	0.5
Current CD8 cell count (cells/mcL), mean (SD)	907.05 (530.46)	947.34 (482.35)	0.2
Current CD4/CD8 ratio, mean (SD)	1.01 (0.54)	1.01 (0.59)	0.6

*WNV* West Nile Virus, *USUV* Usutu virus, *SD* standard deviation; *On stable ART, defined as no treatment discontinuation exceeding one year since ART initiation

In univariate logistic regression analyses (Table 3), geographic origin was strongly associated with WNV and/or USUV virus seropositivity. PLWH originating from Africa (OR 21.22, 95% CI 12.49–36.03), Asia (OR = 23.31, 95% CI: 10.19–53.31), and the Americas (OR 19.16, 95% CI 9.45–38.83) had markedly higher odds of seropositivity (all *p* < 0.001). Male sex was associated with lower odds of seropositivity (OR 0.55, 95% CI 0.37–0.84; *p* = 0.005). While immunological parameters, including CD4 cell count nadir, current CD4 and CD8 cell count, CD8 count, and CD4/CD8 ratio, were not significantly associated with seropositivity, being on stable antiretroviral therapy was associated with increased odds of seropositivity (OR 1.99, 95% CI 1.06–3.47; *p* = 0.021).

**Table 3:** Unadjusted univariate logistic regression analysis of factors associated with WNV or USUV seropositivity

Variable	OR	CI	*P* value
Origin (Africa)	21.22	12.49–36.03	**< 0.001**
Origin (America)	19.16	9.45–38.83	**< 0.001**
Origin (Asia)	23.31	10.19–53.31	**< 0.001**
Origin (Europe)	3.32	1.19–9.30	0.066
Male gender	0.55	0.37–0.84	**0.005**
Age	1.01	0.99–1.04	0.224
CD4 nadir	1.01	0.86–1.20	0.947
Current CD4 cell count	0.89	0.72–1.13	0.412
Current CD8 cell count	0.82	0.62–1.09	0.178
Current CD4/CD8 ratio	1.03	0.71–1.43	0.890
Being On stable ART*	1.99	1.06–3.47	**0.021**
No ART for the last 5 years	3.14	0.89–10.05	0.059
Living in a 2022 WNV-circulating area	0.78	0.50–1.20	0.271

*ART* antiretroviral therapy, *WNV* West Nile Virus; *On stable ART, defined as no treatment discontinuation exceeding one year since ART initiation

Focusing on WNV seropositivity (Table 4), geographic origin remained strongly associated with infection. Participants from Africa (OR 22.06, 95% CI 12.38–39.38), Asia (OR 28.67, 95% CI 12.22–67.26), the Americas (OR 23.61, 95% CI 11.31–49.26), and Europe (OR 4.10, 95% CI 1.43–11.71) had significantly higher odds of WNV seropositivity (all *p* < 0.05). Male sex was again associated with lower odds (OR 0.56, 95% CI 0.36–0.87; *p* = 0.009). No significant associations were observed for CD4 cell count nadir, current CD4 and CD8 cell count, CD4/CD8 ratio, ART exposure, or residence in a 2022 WNV/USUV-circulating area.

**Table 4:** Unadjusted univariate logistic regression analysis of factors associated with WNV seropositivity

Variable	OR	CI	*P* value
Origin (Africa)	22.08	12.38–39.38	**< 0.001**
Origin (America)	23.61	11.31–49.26	**< 0.001**
Origin (Asia)	28.67	12.22–67.26	**< 0.001**
Origin (Europe)	4.10	1.43–11.71	**0.023**
Male gender	0.56	0.36–0.87	**0.009**
Age	1.01	0.99–1.03	0.424
CD4 nadir	0.97	0.82–1.17	0.736
Current CD4 cell count	0.88	0.71–1.12	0.261
Current CD8 cell count	0.79	0.59–1.07	0.200
Current CD4/CD8 ratio	1.01	0.68–1.44	0.977
Being On stable ART	1.97	1.00–3.57	0.059
No ART for the last 5 years	1.78	0.37–6.57	0.415
Living in a 2022 WNV-circulating area	0.76	0.47–1.21	0.261

*ART* antiretroviral therapy, *WNV* West Nile Virus; *On stable ART, defined as no treatment discontinuation exceeding one year since ART initiation

In the multivariable analysis (Table 5), geographic origin remained the only factor independently associated with WNV and/or USUV seropositivity. Compared with participants of Italian origin, the odds of seropositivity were significantly higher among individuals originating from Africa (OR 22.01, 95% CI 12.61–38.41; *p* < 0.001), Asia (OR 21.88, 95% CI 9.27–51.63; *p* < 0.001), the Americas (OR 17.30, 95% CI 8.40–35.65; *p* < 0.001), and Europe (OR 3.49, 95% CI 1.24–9.81; *p* = 0.039). In the sensitivity analysis restricted to participants of Italian origin, none of the variables examined was significantly associated with WNV and/or USUV seropositivity.

**Table 5:** Unadjusted multivariable logistic regression analysis of factors associated with WNV or USUV seropositivity

Variable	OR	CI	*P* value
Origin (Africa)	22.01	12.61–38.41	**< 0.001**
Origin (America)	17.30	8.40–35.65	**< 0.001**
Origin (Asia)	21.88	9.27–51.63	**< 0.001**
Origin (Europe)	3.49	1.24–9.81	**0.039**
Male gender	1.10	0.69–1.74	0.692
Age	1.01	0.99–1.03	0.411
Being On stable ART	1.69	0.90–3.16	0.121

*ART* antiretroviral therapy, *WNV* West Nile Virus; *On stable ART, defined as no treatment discontinuation exceeding one year since ART initiation

## Discussion

In this large cohort of PLWH in Northern Italy, most of whom on stable ART and with undetectable plasmatic HIV viral load, the WNV seroprevalence was 6.2%. Neutralization assays subsequently confirmed WNV-only seropositivity in 86 participants (3.0%), USUV-only seropositivity in 12 (0.6%), and dual WNV/USUV neutralizing antibody positivity in 6 (0.2%). Infections were almost exclusively asymptomatic, occurring predominantly in younger PLWH and individuals of non-Italian origin. No cases of WNND were detected, and no significant association was observed between WNV/USUV seropositivity and immunological parameters. To our knowledge, this is the first study assessing WNV seroprevalence in PLWH, demonstrating a relatively high prevalence compared with reports from similar European settings in blood donors.

Overall WNV IgG seropositivity was 6.2%, with 3.4% confirmed WNV or USUV cases by neutralization assay. Although specific data in PLWH are lacking in the literature, these findings appear higher than those reported in otherwise healthy populations, such as blood donors, in European countries where WNV has become endemic only in recent decades [11, 30, 31]. A nationwide survey in Hungary conducted in 2019 reported a seroprevalence of 4.32% among blood donors [32], while a population-based study in Greece following a lineage 2 epidemic in 2010 found a seroprevalence of 5.8% [33]. Substantially higher seroprevalence rates have been reported in regions where WNV has circulated for longer periods. The highest prevalence described in the literature is 55% among asymptomatic Egyptian blood donors (October 2013–November 2014), with higher rates observed in rural areas and the Nile Delta, consistent with intense and sustained transmission [34]. Elevated seroprevalence has also been documented in other Middle Eastern and North African populations, including 37% in Sudanese individuals and 33% in Egyptians residing in Qatar [35]. Focusing on the most updated literature regarding WNV/USUV seroprevalence in our country, Italian data are limited and mainly derive from blood donors and occupationally exposed populations. A 2023 study of blood donors in southern Italy reported a WNV IgG seroprevalence of 0.32% by ELISA [36], while a nationwide survey conducted in 2021–2022 using ELISA with plaque reduction neutralization test (PRNT) confirmation found WNV seroprevalence rates of 0.66% in blood donors and 1.64% in organ donors, with corresponding USUV seroprevalence rates of 0.90% and 0.82%, respectively [9]. In contrast, a 2023–2024 study of outdoor workers in southeastern Italy using ELISA and neutralization assays reported WNV seroprevalence of up to 6.5% among livestock breeders [37]. Notably, most of these studies did not include neutralization testing, limiting comparability with our analysis, and support the hypothesis that birth outside Italy may be associated with increased odds of WNV or USUV infection. USUV is now established and actively circulating in several European countries, including Italy, Austria, Germany, Hungary, France, Spain, Switzerland, Poland, the United Kingdom, the Czech Republic, Greece, and Belgium, and has also been detected in Israel and parts of Africa [9, 11, 30, 31]. Its geographic expansion remains ongoing [38, 39]. In the Lombardy region, Italy, surveillance data confirm sustained viral circulation in birds, mosquitoes, and humans, with evidence of local evolution and co-circulation of multiple USUV strains [25, 40, 41]. Although higher seroprevalence has been reported in populations with greater mosquito exposure, such as forestry workers, where rates reach 18.1% [42], our findings are comparable to those reported in seroprevalence studies among blood donors in Lombardy, where reported seroprevalence is approximately 1% [42]. In addition, we detected six cases of WNV/USUV dual neutralizing-antibody reactivity, confirming their silent co-circulation [43]. Moreover, their highly homologous envelope proteins result in substantial serological cross-reactivity, posing diagnostic challenges and reinforcing the need for confirmatory neutralization assays [44, 45].

In our cohort, no cases of WNND were observed, and reported symptoms were infrequent and generally mild among PLWH with WNV or USUV seropositivity. Moreover, no definitive association with WNV infection could be established due to the retrospective nature of the study. While severely immunocompromised PLWH are at increased risk of neuroinvasive disease, intensive care admission, and short-term mortality [46, 47], data on PLWH receiving stable ART with sustained HIV viral suppression remain limited. Further studies are required to elucidate the role of NRTIs and NNRTIs the context of arboviral infections in humans. PLWH are now considered to have a life expectancy approaching that of the general population [48]. Accordingly, immune reconstitution likely plays a central role, resulting in clinical manifestations comparable to those observed in immunocompetent individuals. The absence of an association between immunological parameters and WNV or USUV seropositivity in our study may therefore reflect high ART coverage and well-controlled infection, with exposure risk driven primarily by ecological factors rather than host immune status. Although severe neurological manifestations, including meningitis, encephalitis, meningoencephalitis, and atypical presentations such as idiopathic facial paralysis, have been reported [49], the identification, among WNV IgG positive subjects, of 18 USUV infections supports the predominantly asymptomatic or mild clinical course of this infection in our cohort. Experimental data suggest that USUV has a reduced capacity to infect motor neurons compared with WNV, partly due to a more robust intrinsic antiviral response. However, this restriction may be attenuated in immunodeficient states, such as advanced HIV infection, potentially increasing susceptibility to neuroinvasion and motor-related neurological disease [50].

In logistic regression analyses, geographic origin was strongly associated with WNV or USUV seropositivity. Individuals from Africa, Asia, and the Americas had markedly higher odds, suggesting that WNV or USUV seropositivity likely reflects prior exposure in endemic regions rather than infections acquired in Italy. Both WNV and USUV are endemic in several African countries and circulate in parts of Asia and the Americas [51]. In our cohort, the higher WNV seroprevalence observed among migrants compared with Italians may reflect cumulative lifetime exposure. This is further supported by the lack of association between residence in a WNV/USUV-circulating area and increased risk of infection [40, 52]. However, the absolute number of imported WNV cases remains limited compared with other vector-borne diseases (e.g., malaria, dengue, or chikungunya) [53, 54]. Additionally, recent evidence suggests that most WNV infections in Western countries are locally acquired rather than primarily driven by migration from endemic regions [55]. Moreover, in our sample, male sex was associated with lower odds of seropositivity. The literature does not consistently support a protective effect of male sex, as some studies report higher odds of WNV seropositivity among males, such as a large cross-sectional study from Jordan [56], whereas others found no significant sex differences, including recent data from Southeast Europe [57], Tunisia [58], Hungary [32], and Israel [59]. Although occupational exposure is a recognized risk factor for several arboviruses [42], overall, current evidence does not indicate a consistent association between sex and WNV seropositivity, and our finding should therefore be interpreted cautiously. Lastly, immunological parameters—including CD4 cell count nadir, current CD4 cell count, CD8 cell count, and CD4/CD8 ratio—were not significantly associated with seropositivity. Although stable ART was associated with seropositivity in the univariable analysis, this association was not retained in the multivariable model.

This study has several limitations. Its single-center design limits generalizability beyond Northern Italy, and the retrospective nature may have resulted in incomplete ascertainment of exposure history. Although multivariable analyses were performed, residual confounding cannot be excluded, and geographic origin—strongly associated with seropositivity—may reflect migration history. Moreover, the absence of a general population control group precludes direct comparison with people living without HIV. We were unable to distinguish imported from autochthonous infections, and findings may not apply to severely immunocompromised PLWH, as most participants were virologically suppressed with preserved CD4 cell counts. Although the two-step confirmatory algorithm (ELISA followed by IFA, with neutralization performed only on IFA-positive samples) was designed to minimize false-positive results—particularly among borderline ELISA reactions—we cannot exclude that this approach introduced a conservative bias in the estimated seroprevalence. Samples with weak or discordant reactivity that were not confirmed by IFA were not carried forward to neutralization testing, and it is possible that a small proportion of true WNV or USUV infections were not captured by this algorithm. Lastly, although the primary objective of the study was to assess the overall seroprevalence of WNV, IgM testing was performed on IFA-positive samples to provide a presumptive identification of recently acquired infections; unfortunately, it was not possible to perform RT-PCR to confirm active infection in IgG⁺/IgM⁺ positive individuals. Given that a positive serological result does not indicate the exact time of infection, some mild episodes of fever from previous years may not have been reported by patients. Lastly, information on Japanese encephalitis and other flavivirus vaccinations was not available; therefore, we could not assess the potential contribution of vaccine-induced serological cross-reactivity to the observed antibody reactivity. Strengths include the large sample size and the use of confirmatory neutralization assays, minimizing flavivirus cross-reactivity and enabling differential WNV/USUV diagnosis. The cohort is representative of an entire province, as the center is the sole referral hub for HIV care, and the well-characterized population with high ART coverage allows robust assessment in a predominantly well-treated PLWH setting.

## Conclusions

In the context of increasing WNV circulation in Italy, likely driven by climate and ecological changes, this study provides the first assessment of WNV seroprevalence among PLWH. In this largely virologically suppressed, stably treated outpatient cohort, no severe manifestations were observed and symptoms were infrequently recalled. The frequent co-circulation of WNV and USUV further underscores the importance of confirmatory neutralization assays to ensure diagnostic specificity in surveillance and epidemiological studies.

## Supplementary Information

Below is the link to the electronic supplementary material.

Supplementary Material 1

## Data Availability

No datasets were generated or analysed during the current study.
