# Supplementary material for: Prevalence and characterization of West Nile virus infections among people living with HIV in Northern Italy: a sero-repository analysis
Source: Infection. 2026 Aug 11;54(5):2739–48. doi: 10.1007/s15010-026-02919-3 (PMC13727795; doi:10.1007/s15010-026-02919-3)
Supplement: Supplementary file 1 [file 15010_2026_2919_MOESM1_ESM.docx]

**Table S1.** Demographic, clinical, and virological characteristics of PLWH with a WNV IgG⁺/IgM⁺ positive immunofluorescence assay (Acronym: WNV, West Nile Virus; USUV, Usutu virus; SD, standard deviation; *On stable ART, defined as no treatment discontinuation exceeding one year since ART initiation)

| N, (%) | 6 (100) |
| --- | --- |
| Demographics |  |
| Male, n (%) | 4 (67) |
| Age, mean (SD) | 55.7 (± 7.66) |
| Geographic origin |  |
| Italy, n (%) | 4 (67) |
| Africa, n (%) | 1 (17) |
| America, n (%) | 1 (17) |
| Antiretroviral therapy (ART) |  |
| On stable ART*, n (%) | 5 (83) |
| Immunological profile |  |
| CD4 cell count nadir (cells/mcL), mean (SD) | 195.33 (142.77) |
| Current CD4 cell count (cells/mcL), mean (SD) | 937.67 (368.53) |
| Current CD8 cell count (cells/mcL), mean (SD) | 862.17 (201.09) |
| Current CD4/CD8 ratio, mean (SD) | 1.09 (0.38) |
| Anamnestic data and symptoms among confirmed WNV or USUV cases (*) | |
| History of travel (self-reported), n (%) | 2 (33) |
| Symptoms reported during travel, n (%) | 0 (0) |
| Symptoms reported during summer, n (%) | 0 (0) |
| Neutralization assays results |  |
| Confirmed WNV cases, n (%) | 6 (100) |
| WNV positive, n (%) | 4 (67) |
| USUV positive, n (%) | 2 (33) |

**Figure S1.** Flow diagram (Figure S1) summarizing the testing algorithm and sample numbers at each step (Acronym: ELISA, Enzyme-Linked Immunosorbent Assay, IFA, Indirect Immunofluorescence Assay; WNV, West Nile Virus; USUV, Usutu virus)
